# The Institutional Worker

**Published:** 1912-04-13

**Authors:** 


THE
### INSTITUTIONAL WORKER
**Being a Special Supplement to "The Hospital"**

### Editor's Notices.

**Contributions:**

Contributions should be written, or preferably typed, on one side of the paper only, and all articles sent in are accepted upon the distinct understanding that they are forwarded to The Hospital only.

The Editor cannot undertake to return MSS. not used, but when a stamped directed envelope is enclosed the MSS. may be returned if a special request is made.

Accepted articles and paragraphs of news will be paid for after publication at the scale rate.

**Address:**

To prevent delay all contributions and letters on editorial business must be addressed exclusively to the Editor, "The Hospital," 29 Southampton Street, Strand, London, W.C. It is important that this regulation shall be strictly observed.

**Correspondence:**

Correspondence on all subjects is invited. The name and address of correspondents must be given as a guarantee of good faith, but not necessarily for publication.

**Special Articles:**

Special articles are invited, and questions, inquiries, and paragraphs upon all matters relating to the work, administration, and management of general, special, mental, fever, cottage and convalescent hospitals, sanatoria, homes, institutions, societies and organisations for the treatment or care of the sick, injured and dependants of all classes. Every matter affecting the interests and welfare of the staff of all grades working in these institutions will receive special consideration.

**Administrative Medicine, Research and Items of News:**

Special terms will be paid for approved articles on subjects in this wide field by experts who have made some section of it their special study and interest. We wish to give prominence to every movement tending to advance accuracy of diagnosis, efficiency in treatment, and the development of modern methods to eradicate disease and promote the welfare of the suffering.

**A Bureau of Information:**

We invite inquiries and applications for information and help in providing for that numerous class of sufferers who require special care or house-room which they cannot obtain in their own homes or secure for themselves. We cannot, however, prescribe, or recommend practitioners.

**Books for Review:**

Publishers are particularly requested to send advance proofs of any new books of importance whenever possible, as the Editor has made arrangements to publish immediate reviews on a new plan.

**Photographs, Plans, Blocks, and Illustrations:**

It is requested that wherever possible MSS. may be accompanied by illustrations in any of the above forms. The name of the sender and of the article to which it belongs should be written on the back of each photograph, drawing, or block for purposes of identification.

**Local Papers:**

Newspapers containing reports or news paragraphs should be marked and addressed to the Sub-Editor.

**The Coupon System:**

A coupon will be found at the bottom of the third inside page of the cover each week. This coupon must be attached to every question or inquiry to which an answer is desired in The Hospital.

### The Things that Help.

There are many ways in which our readers may assist us in our purpose to render The Hospital of the greatest possible utility to all interested or engaged in Institutional work. For instance, some of your colleagues and friends may yet be unaware of the important developments that have been effected in this Journal within the past few months, and how essential The Hospital now is to everyone connected or associated with Charitable, Poor Law, and Municipal Administration and Work. Consequently, it would be an act of kindness to direct their attention to the Journal, and to point out to them the advantages of securing a copy of The Hospital every week. Again, "The Institutional Worker" section contains a large number of Appointments Vacant, and our readers will help themselves and assist us in making this portion of greater value by mentioning The Hospital when replying to an announcement which appears in its columns. It is our aim to make "The Institutional Worker" a complete record of all Vacancies that occur from time to time in every department of Institutional Life, and with this view Special Rates are allowed to the Authorities of Institutions who agree for one year to send all their Public Notices for insertion in The Hospital. Inquiries are cordially invited from Executive Officials, and will receive our prompt and careful attention.

### Manager's Notices.

**All letters relating to Subscriptions, Advertisements, and other business matters must be addressed to The Manager, "The Hospital," The Hospital Building, 28 and 29 Southampton Street London, W.C.**

**Advertisements:**

To ensure insertion, all advertisements for The Hospital must reach the Manager not later than Wednesday morning in each week. For scale of charges see pages v and 2.

**Subscriptions** may begin at any time, and are payable in advance. Cheques and Post-Office orders should be crossed London County and Westminster Bank, Covent Garden branch, and made payable to the Manager of The Hospital.

Rates of Subscription (payable in advance).

| | |
|---|---|
| UNITED KINGDOM. | |
| Three Months (including Postage) ... ... ... ... | 2s. 0d. |
| Six Months do. ... ... ... ... | 4s. 0d. |
| Twelve Months do. ... ... ... ... | 6s. 6d. |
| FOREIGN AND COLONIAL. | |
| Three Months (including Postage) ... ... ... ... | 2s. 6d. |
| Six Months do. ... ... ... ... | 5s. 0d. |
| Twelve Months do. ... ... ... ... | 8s. 8d. |

*SUBSCRIPTION FORM.*

*Please send me* "The Hospital" *for months, commencing with your issue of.........................., for which please find enclosed..........................*

*Name.........................................*

*Address.........................................*

.........................................

Date.........................................

*To..............................Newsagent,*

**Remittances:**

**It is especially requested that remittances be made by cheque or Postal Order, and in halfpenny stamps only when the amount is under sixpence.**

### OFFICIAL APPOINTMENTS VACANT.

### Poor-Law Vacancies.

Assistant Cook (£24), Bermondsey Guardians. Matron of the Workhouse, Ladywell Road, Lewisham, S.E. April 25.

Assistant Nurse (£25), Atcham Union. E. P. Everest. C. to G., Atcham Union Offices, St. John's Hill, Shrewsbury. April 22.

Assistant Nursery Attendant (£18), Lewisham Union. Mr. H. C. Mott, C. to G., 394 Lewisham High Road, S.E. April 13.

Assistant Nurse (£20), Skipton Union. Mr. M. R. Knowles, C. to G., Skipton. April 16.

Charge Nurse (£35), Bromley Union. E. Haslehurst, C. to G., Union Offices, Park House, Bromley, Kent. April 15.

Charge Nurses (£30), Chesterfield Union. R. F. Hartwright. C. to G. Union Offices, Chesterfield. April 19.

Charge Nurses (£30), Plymouth Guardians. Mr. W. H. Davy, C. to G., Plymouth. April 16.

Engineer (35s. weekly), Bermondsey Guardians. Mr. E. Pitts Fenton, C. to G., 283 Tooley Street, S.E. April 24.

Female Attendant (£20), Windsor Union. Mr. E. C. Durant, Acting C. to G., Windsor. April 13.

Female Lunatic Attendant (£18), Cardiff Union. Mr. A. J. Harris, C. to G., Cardiff. April 18.

Head Nurse (£32), Camberwell Guardians. Mr. C. S. Stevens, C. to G., Peckham Road, S.E. April 17.

Head Nurse (£35), Redruth Union. T. Peter. C. to G. Union Offices, Redruth. April 25.

Infant Protection Visitor, etc. (£52), Richmond Union. Mr. P. Umney, C. to G., Richmond, Surrey. April 25.

Labour Master (£34), Croydon Union. Mr. H. List, C. to G., Mayday Road, Thornton Heath, Surrey. April 19.

Male Night Nurse (27s. weekly, non-resident), Cardiff Union. Mr. A. J. Harris, C. to G., Cardiff. April 18.

Master's General Assistant and Storekeeper (£25), Chelmsford Union. Mr. A. S. Duffield, C. to G., Chelmsford. April 22.

Night Nurse (£27 10s.), Battle Union. Mr. F. G. Ticehurst, C. to G., Battle. April 18.

Nurse (£30), Abergavenny Union. Mr. W. H. P. Scanlon, C. to G., Abergavenny. April 17.

Nurse (£30), Skipton Union. Mr. M. R. Knowles, C. to G., Skipton. April 16.

Porter and Portress (married couple) (£30 and £20), Wandsworth Union. Mr. F. W. Piper, C. to G., St. John's Hill, Wandsworth. April 24.

Porter and Superintendent of Labour (£25), Bradfield Union. Mr. R. E. Rawstorne, C. to G., 4 Bridge Street, Caversham, Reading. April 15.

Probationer Nurses (£10), Burton-on-Trent Union. C. F. Chamberlin, C. to G., Union Offices, Burton-on-Trent. April 20.

Probationer Nurse (£10), Tonbridge Union. N. R. Stone, C. to G., 23 Church Road, Tunbridge Wells. May 2.

Probationer Nurses (£10), Wandsworth Union. Matron, The Infirmary, St. John's Hill, Wandsworth, S.W.

Probationer Nurses (£13), West Ham Union. T. Smith, C. to G., Union Road, Leytonstone, N.E.

Probationers (4) (£10), Gateshead Union. Mr. G. Craighill, C. to G., Gateshead. April 16.

Relieving Officer (£120), Bedwellty Union. Mr. H. J. C. Shepard, C. to G., Tredegar. April 22.

Relieving Officer, etc., Pontypool Union. Mr. T. Watkins, C. to G., Pontypool. April 16.

Staff Nurse (£22), Camberwell Guardians. Mr. C. S. Stevens, C. to G., Peckham Road, S.E. April 18.

Superintendent Nurse (£60), Coventry Union. Mr. E. A. Evans, C. to G., Coventry. April 16.

### Institutional and Administrative Vacancies.

Matron (£100), City of London Lying-in Hospital. R. A. Owthwaite, Secretary. April 15.

Matron (£45), Ilkeston Hospital. A. J. Worrall, Secretary, 18 Drummond Road, Ilkeston. April 17.

Sister Housekeeper (£45), Royal Sussex County Hospital, Brighton. R. B. Jay, Secretary. April 23.

### Municipal Vacancies.

Assistant Chief Constable (£600), Lancs. Standing Joint Committee. Mr. H. E. Clare, Clerk of the Peace, Preston, April 15.

Accounts Clerk (£80), Yeovil T.C. Mr. A. B. Barrand, Borough Accountant, Yeovil. April 13.

Assistant Sanitary Inspector (£100), Chiswick U.D.C. Mr. E. F. Collins, Clerk to the Council, Town Hall, Chiswick. April 17.

Borough Surveyor (£250), Newark T.C. Mr. G. Tallents, Town Clerk, Newark. April 20.

Chief Constable (£400), Preston T.C. Mr. A. Howarth, Town Clerk, Preston. April 15.

Clerk to Council (£120), Tilbury U.D.C. Mr. J. Beck, 2 Orsett Road, Grays. April 13.

Collector (£80), Tilbury U.D.C. Mr. J. Beck, 2 Orsett Road, Grays. April 13.

Commercial Assistant for Electricity Department (£100), Gillingham T.C. Mr. F. C. Boucher, Town Clerk, Gillingham, Kent. April 22.

Coroner (£1,150), L.C.C. The Clerk to the Council, Spring Gardens, S.W. April 22.

Inspector of Nuisances (£120), Tilbury U.D.C. Mr. J. Beck, 2 Orsett Road, Grays. April 13.

Inspector of Nuisances, etc. (£65), Newent R.D.C. Mr. C. Tunnicliff, Clerk to the Council, Newent. April 17.

Inspector of Weights and Measures (£175), East Sussex C.C. Mr. F. Merrifield, Clerk to the Council, Lewes. April 19.

Inspector under Shops Act, etc. (£120), Ealing T.C. Mr. G. E. Brydes, Town Clerk, Ealing. April 22.

Matron (£105), Horton Asylum. Mr. H. F. Keene, Clerk to the Asylums Committee, 6 Waterloo Place, S.W. April 18.

Meat and Dairy Inspector (£120), Rochdale T.C. Mr. W. H. Hickson, Town Clerk, Rochdale. April 18.

Midwifery Nurse (£100), Manchester T.C. Mr. T. Hudson, Town Clerk, Manchester. April 13.

Road Foreman (50s. weekly), Lambeth B.C. Mr. H. J. Smith, Town Clerk, Brixton Hill, S.W. April 18.

Sanitary Inspector (£150), Lewisham B.C. Mr. E. Wright, Town Clerk, Catford, S.E. April 13.

Sewage Farm Sub-Manager (£100), Coventry T.C. Mr. G. Sutton, Town Clerk, Coventry. April 15.

Sewers Foreman (£3 weekly), Lambeth B.C. Mr. H. J. Smith, Town Clerk, Brixton Hill, S.W. April 18.

Superintendent Nurse (£42), M.A.B. The Clerk to the Board, Embankment, E.C. April 16.

Surveyor (£208), Tilbury U.D.C. Mr. J. Beck, 2 Orsett Road, Grays. April 13.

Surveyor, etc. (£156), Irlam U.D.C. Mr. J. Cooke, Clerk to the Council, Irlam. April 16.

### Miscellaneous Vacancies.

Resident Nurse (£60), H.M.S. *Conway*, Commander H. W. Broadbent, R.N.R. H.M.S. *Conway*, Rock Ferry, Cheshire. April 15.

Woman Inspector for Domestic Subjects (£220), West Riding Education Committee. Education Department, County Hall, Wakefield. April 18.

### APPOINTMENTS, RESIGNATIONS, &c.

### Poor Law Appointments.

Bentham, Mr. T. H., Assistant Clerk, Preston Union.

Blackwell, Miss F. E., Assistant Scholmistress, South Manchester Township.

Brown, Mr. J. D., Schoolmaster at the Aldersbrook Receiving Homes, West Ham Union.

Chatburn, Mrs. C. J., Assistant Schoolmistress, Salford Union.

Collins, Mr. W. G., Second Assistant Clerk, Dartford Union.

Dalton, Mr. W., Assistant Clerk, Dartford Union.

Evans, Mr. B., Assistant Clerk, Bangor and Beaumaris Union.

Fulcher, Miss A. M., Matron of the Workhouse, Atcham Union.

Goldsworthy, Miss M. N., Midwifery Pupil, Poplar Borough Parish.

Green, Miss E. A., Night Superintendent Nurse, Stockport Union.

Hancock, Miss E. D., Matron of the Downs School, Metropolitan Asylum District.

Legat, Miss E. M. C., Matron of the Sanatorium, South Manchester Township.

Peers, Miss V. A., Schoolmistress, New Forest Union.

Spelman, Mr. C. H., re-appointed Master of the Workhouse, Wirral Union.

Spelman, Mrs. E. M. St. C., Matron of the Workhouse, Wirral Union.

Townend, Mr. J., Clerk to the Guardians, Selby Union.

Sinclair, Mr. E. D., Second Class Junior Clerk, Hackney Union.

### Charge Nurses.

Howard, Miss E., Oldham Union.

Hunter, Miss A., Morpeth Union.

MacSweeney, Miss S., Hampstead Parish.

Pitt, Miss A. V., East Preston Union.

### Nurses.

Allan, Miss H. L., Hackney Union.

Cassanet, Miss E. F., Tendring Union.

